# Pomegranate peel-derived Cu/hydrochar nanocomposites: a sustainable catalyst for biodiesel production via oleic acid esterification

**DOI:** 10.1186/s13065-026-01885-z

**Published:** 2026-10-06

**Authors:** Amal A. Alkahlawy, Delvin Aman, Ahmed A. Galhoum, Tamer Zaki

**Affiliations:** 1 Petroleum Refining Department, Egyptian Petroleum Research Institute (EPRI), 1 Ahmed El Zomour St., PO Box 11727, Nasr City, Cairo, Egypt; 2 Nuclear Materials Authority, P.O. Box 530, El-Maadi, Cairo, Egypt

**Keywords:** Pomegranate peel, Hydrothermal treatment, Cu/hydrochar nanocomposite, Biodiesel, Catalytic esterification, Oleic acid

## Abstract

Transforming pomegranate peel waste into a biodiesel catalyst advances waste-to-resource circularity. A facile hydrothermal carbothermal reduction method was used to synthesize copper-decorated hydrochar nanocomposites derived from peel bio-waste and CuSO_4_, with Cu(II) concentrations of 10, 25, and 50 mmol/L (denoted as Cu10/HC, Cu25/HC, and Cu50/HC, respectively). The physicochemical properties of the nanocomposites were characterized by FTIR, XRD, BET, XPS, SEM-EDX, and NH₃-TPD, and their catalytic performance was evaluated for biodiesel production via the esterification of oleic acid (OA) with ethanol. Structural analysis confirmed highly pure, crystalline zero-valent copper (Cu⁰) with an FCC nanostructure and spherical morphology (average size ~ 25–63 nm), well-dispersed throughout the hydrochar-derived carbon matrix. XPS verified oxygen functionalities (–COO⁻, > C = O, C–OH) alongside Cu⁰ on the nanocomposite surfaces. Moreover, textural analysis revealed that Cu25/HC exhibited the highest surface area (29.6 m²/g) and pore volume (0.099 cm³/g), whereas Cu50/HC showed reduced porosity but introduced significant acidity (54.9 µmol/g, according to NH_3_-TPD). Catalytic efficiency increased with higher Cu dispersion density (Cu atoms/nm^2^; R² =0.996): Cu50/HC achieved the best performance (97.4% conversion), followed by Cu25/HC (96.6%) and Cu10/HC (96.0%), outperforming bulk Cu content and BET surface area. Under optimal conditions (70 °C, 5 h, 1.0 wt% catalyst relative to OA, and an ethanol/OA molar ratio of 15:1) the Cu50/HC catalyst attained complete OA conversion. Thermodynamic parameters were determined and indicated an endergonic and thermodynamically non-spontaneous reaction. Notably, the catalyst retained 97.1% of its initial activity after six consecutive cycles, demonstrating excellent operational stability, as confirmed by the FTIR, XRD, and Cu content. These findings position the Cu50/HC nanocomposite as a cost-effective, sustainable, and efficient catalyst for biodiesel production.

**Supplementary Information:**

The online version contains supplementary material available at https://doi.org/10.1186/s13065-026-01885-z.

## Introduction

Fossil fuels currently supply approximately 80% of global energy; however, declining reserves and price volatility underscore the urgent need for sustainable alternatives. Among these, biomass-derived biodiesel has emerged as a cleaner, carbon-neutral substitute for petrodiesel [1–5]. Biodiesel, primarily composed of fatty acid methyl esters (FAME) or fatty acid ethyl esters (FAEE), is typically produced via the esterification or transesterification of animal and vegetable oils with methanol or ethanol in the presence of acidic or basic catalysts [6, 7]. In parallel with socioeconomic development, rising energy demand and environmental pollution have intensified research into eco-friendly biodiesel production methods, including the use of biomass waste as catalyst precursors [8, 9]. Catalytic reactions are broadly classified into homogeneous and heterogeneous types based on the catalyst employed. Heterogeneous catalysis has gained increasing research interest due to its distinct advantages, including straightforward procedures, ease of product separation, and reduced environmental impact. Common heterogeneous catalysts include inorganic acid salts, solid heteropoly acids, and metal oxides [9–13].

Biomass waste, including agricultural residues and sewage sludge, is crucial for generating valuable products in a bioeconomy. Agricultural waste from fruit production accounts for 10–60% by weight, posing environmental challenges [14]. Global food waste costs approximately one trillion USD annually, contributing 8–10% of greenhouse gas emissions [15]. Inedible agricultural waste can be converted into biochar through pyrolysis, combustion, gasification, and thermal/hydrothermal carbonization (HTC) processes [16, 17]. Notably, HTC efficiently converts wet biomass into hydrochar without pre-drying, yielding superior physicochemical properties compared to conventional biochar. Furthermore, HTC enables the preparation of metal or metal oxide nanocomposites via a simple in-situ carbonization method, producing nanocomposites with enhanced thermal and chemical stability [18–20].

Nanocomposites, which contain at least one nanoscale phase, synergistically combine the properties of their constituent components. They are broadly classified into carbon-based, metal-based, and polymer-based nanocomposites. Carbon-based variants include graphene, carbon nanotubes, and carbon nanofibers [21–23]. Metallic nanostructures, particularly those of Pt, Au, Cu, and Ag, exhibit exceptional electronic and catalytic properties, rendering them valuable for energy and environmental applications [21, 24–26]. Among these, copper nanoparticles (Cu⁰) offer a compelling alternative to noble metals (e.g., Ag, Au, Pd) due to their low cost, natural abundance, and high redox activity [27–30]. Additionally, nanomaterials provide exceptionally high specific surface area, and abundant active sites for catalytic reactions, along with superior mechanical and thermal stability, which are especially advantageous for biomass-derived catalysts like hydrochar, directly improving triglyceride conversion efficiency in biodiesel production [6, 8, 31, 32]. In the context of biodiesel production, Cu⁰ has demonstrated high catalytic efficiency in converting soybean and palm oil to biodiesel [2, 33], as well as moderate catalytic activity in converting lignin into high-quality bio-oil [34]. Similarly, copper(II) oxide (CuO) has shown good catalytic activity in biodiesel production from sunflower, castor, and waste cooking oils [35–37]. Supported metallic nanostructures on mesoporous, oxygen-rich hydrochar derived from agro-waste via an in-situ reaction yield a hybrid material that combines adsorption, catalysis, and redox activity [26]. The hydrochar matrix prevents Cu⁰ aggregation and enhances reactant binding via oxygen groups, while CuNPs enable rapid electron transfer and transesterification [25, 27, 28, 38–40]. Although prior syntheses of CuNPs using biopolymers or synthetic surfactants [41, 42], Cu-based catalysts for efficient biodiesel preparation remain rarely reported.

This study presents a sustainable, surfactant-free and external-reductant-free synthesis of mesoporous Cu⁰/hydrochar nanocomposites via in-situ hydrothermal carbonization of pomegranate peel waste for oleic acid esterification, which serves as both carbon source and reducing agent. The catalyst combines Lewis and Brønsted acid sites, achieving complete oleic acid conversion with only 1 wt% catalyst loading. To our knowledge, this is the first report utilizing pomegranate peel-derived hydrochar as a support for zero-valent copper nanoparticles in oleic acid esterification for biodiesel production. Unlike previous approaches, our one-pot method simultaneously carbonizes the biomass and reduces Cu(II) to Cu⁰, leveraging the peel’s native reducing capacity. While carbon-supported Cu catalysts have been explored elsewhere, their systematic evaluation in esterification with full kinetic and thermodynamic characterization remains scarce. In addition, comprehensive kinetic and thermodynamic analyses provide new insights into the catalytic mechanism and reaction behavior. Accordingly, this study aimed to: (a) synthesize and characterize Cu⁰/HC nanocomposites using SEM, FTIR, NH₃-TPD, and XPS; (b) optimize esterification parameters (catalyst loading (0.5–2 wt%), ethanol/oleic acid molar ratio (5:1–15:1), time (1–9 h), and temperature (60–80 °C)) and determine thermodynamic parameters; and (c) evaluate catalyst reusability and stability.

## Experiments

### Hydrothermal synthesis of Cu/hydrochar nanocomposites

Initially, pomegranate peels were collected from a local household or market in New Cairo City, Egypt. The peels were thoroughly washed with deionized water, dried at 85 °C for 12 h, ground, and sieved to a particle size of less than 53 μm before being stored in a desiccator for future use. The Cu/hydrochar (HC) nanocomposites were synthesized using a one-pot hydrothermal reduction method. Specifically, 3.0 g of pomegranate peel powder was dispersed in 75 mL of CuSO_4_·5H_2_O solution (Alpha Chemika, India) at concentrations of 10, 25, and 50 mM. The dispersion process was carried out using ultrasonication for 45 min to ensure a homogeneous mixture before transferring the solution into a Teflon-lined stainless-steel autoclave with a capacity of 100 cm³. The sealed autoclave was maintained at 175 °C for 18 h. After naturally cooling to room temperature, the brown powders were obtained and thoroughly washed with deionized water and ethanol. The products were then dried at room temperature (28 °C) for 24 h and labeled as Cu10/HC, Cu25/HC, and Cu50/HC, respectively. For comparison, hydrochar (HC) was prepared in the absence of copper precursors. The product is labeled as r-PG.

### Characterization

The FTIR spectra were examined using a Thermo-Fisher Nicolet IS10 spectrometer (Waltham, MA, USA). X-ray diffraction (XRD) data was acquired with a Bruker AXS-D8 Advance XRD spectrometer (Karlsruhe, Germany) employing Cu Kα radiation (k = 1.5405 Å). Acidity measurements were conducted through temperature-programmed desorption (NH_3_-TPD) using an automated BEL CAT instrument (MicrotracBEL Corp., Osaka, Japan). The surface area was assessed using the BET method (N2-adsorption isotherms via Quantachrome TouchWin™, version 1.2, Boynton Beach, FL, USA) at 80 °C (degassing temperature) for 24 h, while pore volume was determined using the BJH technique. X-ray photoelectron spectroscopy (XPS) data was collected with a Thermo Fisher K-Alpha spectrometer (Waltham, USA) using an AlKα-radiation source (200 W). A scanning electron microscope (SEM) with energy-dispersive X-ray analysis (EDX), Quanta FEG 250 SEM-EDX, was also employed for analysis.

### Catalytic studies

The catalytic esterification of oleic acid with ethanol was performed in a 100 mL glass reactor equipped with a magnetic stirrer and a condenser, controlled by a programmable thermostat and placed in an oil bath. Batch experiments were conducted using a constant dose of nanocomposites (ranging from 0.5 to 2.0 wt%), which were added to the ethanol/oleic acid mixture at molar ratios between 5:1, 10:1, and 15:1. The reactions were carried out at specific temperatures (60, 70, and 80 °C) for varying durations (1–9 h). To monitor the conversion of oleic acid to ester, 5 mL samples were collected at 60 -minute intervals. The catalytic residue and water produced during the esterification process were separated using centrifugation at 5000 rpm for 5 min, and the samples were titrated against a 0.1 M KOH solution, as illustrated in Eq. (1).

1 $$\:\mathrm{A}\mathrm{c}\mathrm{i}\mathrm{d}\:\mathrm{v}\mathrm{a}\mathrm{l}\mathrm{u}\mathrm{e}\:=\frac{V\:\times\:0.1\times\:56.1}{M}$$

here V and M represent the consumed volume of KOH and the mass of oleic acid, respectively; the values 0.1 and 56.1 denote the concentration and molar mass of KOH. Oleic acid conversion was determined based on the change in acid value using Eq. (2):

2 $$\:Conversion\:\left( \% \right)\: = \frac{{(AV_{0} \: -\:AV_{t}) \times100}}{{AV_{0} }}$$

In the equation, AV_0_ and AV_t_ represent the acid value of the fatty acid at the initial time (t = 0) and at equilibrium (t = t) after the reaction, respectively. All reported values represent the means of duplicate measurements, and some selected are triplicated measurements, with a standard deviation (SD) of approximately ± 3–5% (i.e., Mean ± SD).

## Results and discussion

### Synthesis and characterization

#### Synthesis mechanism

A cost-effective carbothermal reduction method was developed for the in-situ synthesis of Cu/HC nanocomposites, utilizing pomegranate peels as both reducing and stabilizing agents under high-temperature and high-pressure conditions within a sealed reactor [43]. In this process, Cu(II) ions were deposited onto a hydrochar (HC) matrix, resulting in a homogeneous solid-phase product [44]. The key mechanistic steps include the following: elevated hydroxide ion concentration [OH⁻], arising from the increased ionic product of water (Kw), accelerates the hydrolysis of Cu(II) ions; the reduced dielectric constant and lower solvating power of water under these conditions promote the precipitation of Cu(OH)₂, thereby facilitating the formation of nucleation sites for nanoparticle development; the application of high pressure and temperature induces the dehydration of Cu(OH)₂, yielding CuO; at 180 °C, the water–gas shift reaction (CO + H₂O ↔ CO₂ + H₂) generates a reducing atmosphere, facilitating the reduction of CuO to Cu⁰ nanoparticles (Cu⁰NPs) via H₂; alternatively, the decomposition of biomass produces hydrochar and acidic species, which can form copper format, subsequently leading to the generation of Cu⁰NPs under supercritical conditions through the decomposition of formic acid [45].

The EDX results (Fig. S1) corroborate the quantitative atomic absorption spectroscopy (AAS) analysis, confirming the successful incorporation of copper into the hydrochar matrix as precursor concentration increases. The observed trend agrees well with the theoretical copper contents estimated from the hydrochar yield (51.9%) under the applied synthesis conditions. The Cu signal increased progressively from 5.7 wt% (1.2 at%) for Cu10/HC to 17.5 wt% (4.1 at%) for Cu25/HC and 26.2 wt% (6.6 at%) for Cu50/HC, demonstrating enhanced copper immobilization with increasing CuSO₄ concentration. Concurrently, the oxygen content decreased from 27.6 to 14.1 wt%, indicating the involvement of oxygen-containing functional groups in Cu²⁺ complexation and subsequent Cu–O bond formation during hydrothermal carbonization. Notably, the Cu contents determined by EDX (expressed as wt%, corresponding to the measured weight percentages) are considerably higher than the expected bulk AAS values of 2.6 ± 0.2, 5.5 ± 0.4, and 8.1 ± 0.3 wt% for Cu10/HC, Cu25/HC, and Cu50/HC, respectively. This difference is anticipated because EDX is a surface-sensitive technique that preferentially analyzes copper-enriched surface regions, whereas AAS quantifies the average copper concentration throughout the entire hydrochar matrix. Therefore, the complementary EDX and AAS results consistently demonstrate the progressive enrichment and effective immobilization of copper within the hydrochar framework.

#### SEM-EDX analysis

**Fig. 1:**
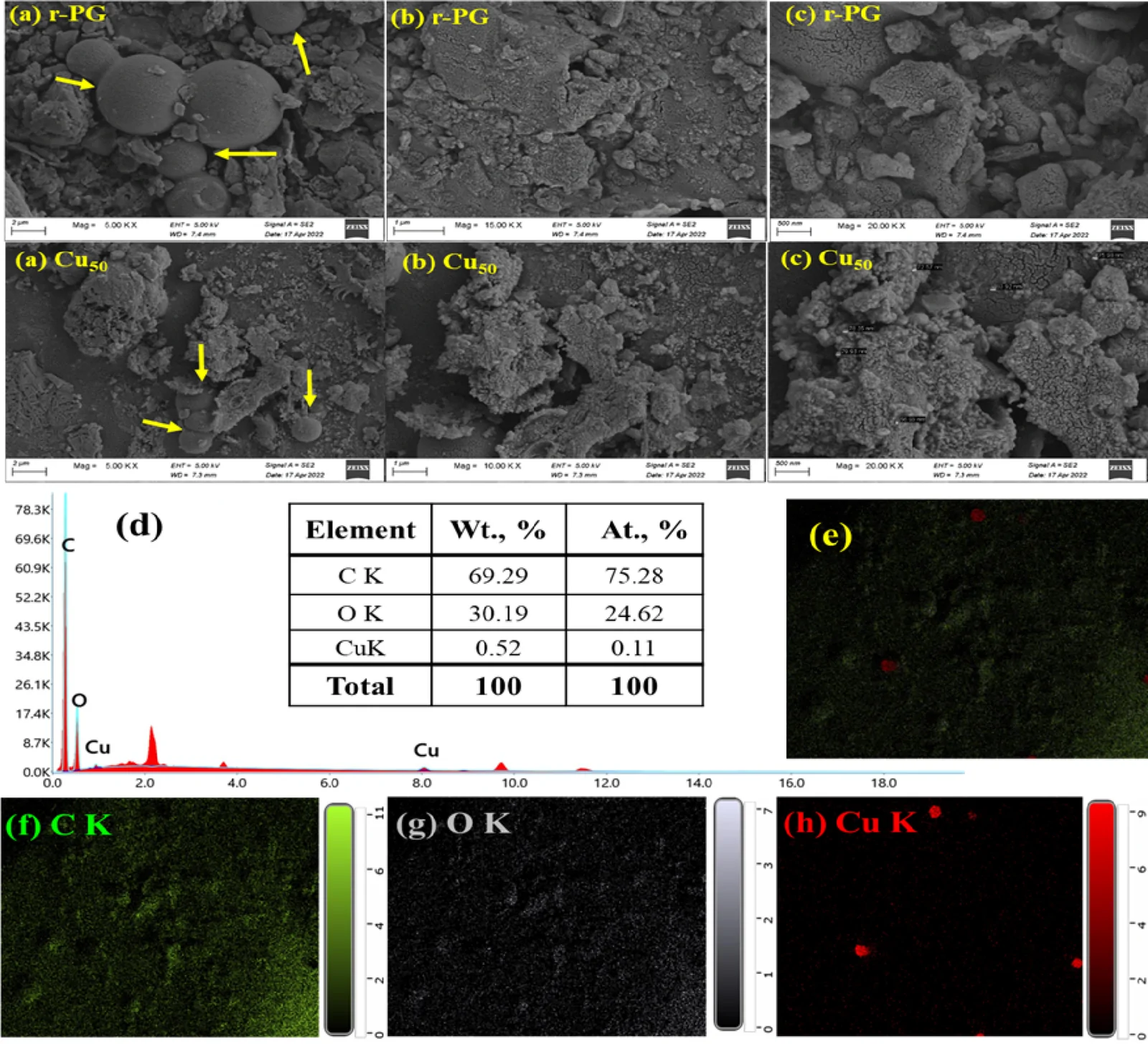
SEM images of r-PG and Cu50/HC at magnifications: (**a**) 5000×, (**b**) 10,000× and (**c**) 20,000×. EDX analysis of Cu50/HC (**d**), color-SEM-EDX mapping analysis (**e**), and elemental mapping (**f**–**h**)

Figure 1 shows SEM images revealing the microstructure of r-PG and the Cu50/HC nanocomposite. The SEM imaging of r-PG (Fig. 1a–c, upper panel) reveals relatively smooth, well-defined spherical particles ranging from submicron dimensions to approximately 3 μm, densely packed into a heterogeneous granular texture. Following copper loading, the Cu50 nanocomposite (lower panel) exhibits pronounced morphological changes, including rougher, irregular surfaces with fractured domains and porous features. Arrows in Fig. 1a indicate copper-containing phases anchored onto the carbonaceous matrix. At higher magnifications (10000–20000×), a hierarchical architecture emerges, comprising aggregated particles with protrusions and surface irregularities. Fine granular features confirm successful nucleation of copper species, while interparticle voids and micro-cavities suggest secondary porosity. This enhanced roughness and porosity promote active site accessibility and mass transfer, favoring catalytic applications.

The EDX spectroscopy (Fig. 1d) confirms the presence of C, O, and Cu elements, as indicated by the C Kα signal at 0.276 keV and the O Kα1 signal at 0.523 keV. The Cu signals show distinct peaks, including the Kα Cu signal at approximately 8.040 keV and a faint Lα peak at around 0.930 keV [46]. The Cu50/HC nanocomposite is predominantly carbon (69.29 wt%) and oxygen (30.19 wt%), with oxygen originating from surface functional groups (e.g., hydroxyl, carboxyl) that aid metal immobilization. Additionally, the low Cu content (0.52 wt%) indicates fine dispersion without large formation of Cu-rich domains. Furthermore, SEM-EDX color mapping and elemental distribution maps (Fig. 1e–h) confirm homogeneous C, O, and Cu signals on the copper/hydrochar nanocomposite surface. Notably, the Cu map (Fig. 1h) shows well-dispersed copper signals without segregation, indicating successful immobilization of copper species onto the hydrochar matrix during synthesis.

#### FTIR spectrometry

**Fig. 2:**
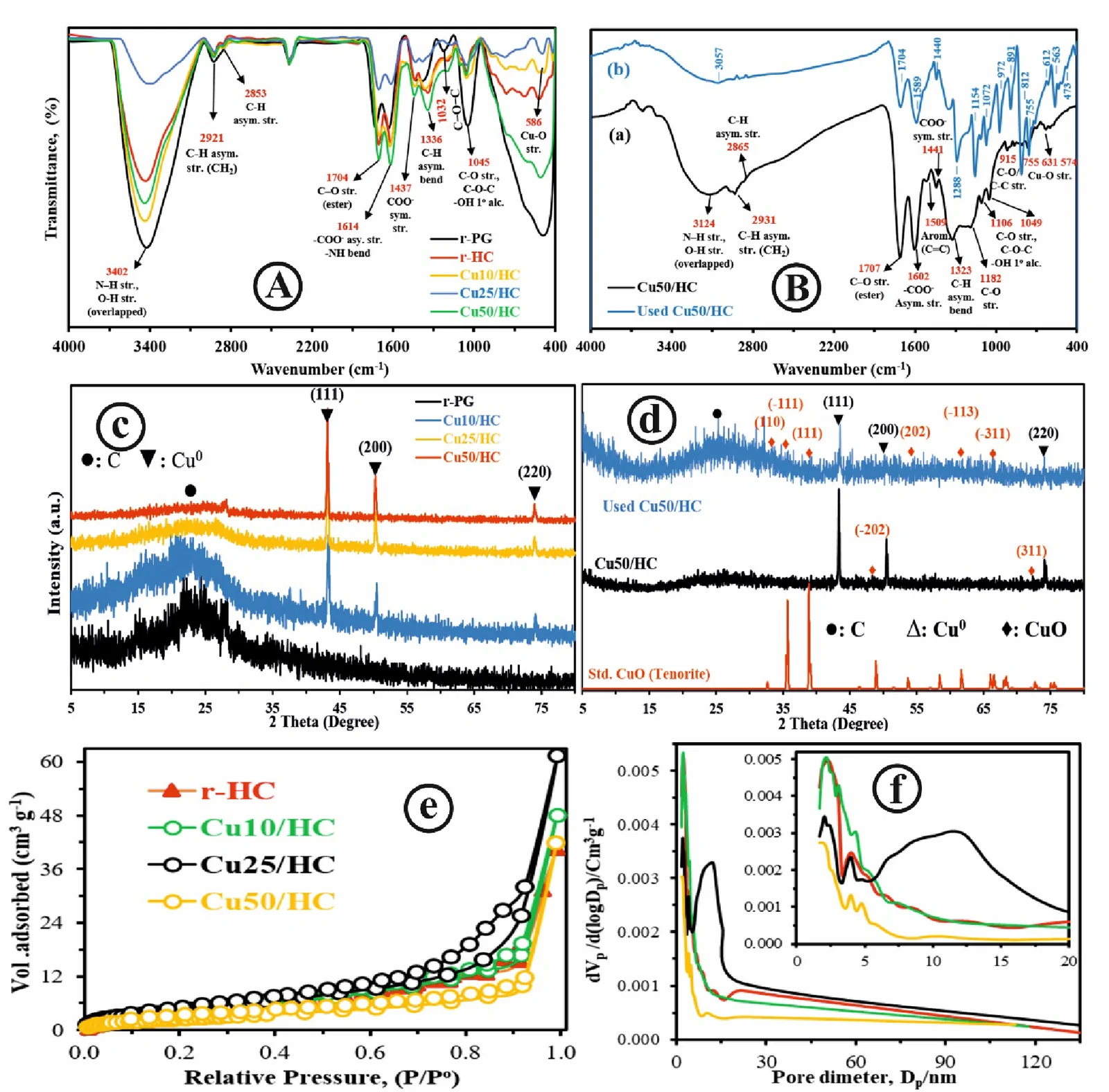
FTIR spectra (**A**, **B**) and XRD patterns (**C**, **D**) of r-PG, Cu/HC nanocomposites, and fresh/spent Cu50/HC, respectively; and textural characterization of r-HC, Cu10/HC, Cu25/HC, and Cu50/HC–BET surface analysis (**e**) and pore size analysis (**f**)

Figure 2 (and Table S1) present the FTIR spectra of pristine PG peel powder, hydrothermally treated r-PG, and Cu⁰-loaded samples, revealing several common bands. The pristine PG powder shows a broad absorption band at 3393–3429 cm⁻¹, attributed to overlapping –OH (and trace H_2_O), –NH stretching vibrations, and O-H stretching related to polysaccharide hydrogen bonds [43–44]. High-intensity peaks at 2918–2923 cm⁻¹, 2849–2856 cm⁻¹, and 1339–1378 cm⁻¹ correspond to C − H stretching (CH₂, symmetric and asymmetric) and C–H bending [43, 45–46]. A peak around 2363 cm⁻¹ is likely due to O = C=O stretching in CO₂ [45], while the peak at 1628 –1613 cm⁻¹ indicates N − H bending vibrations (–NH₂ and > NH) and symmetric stretching of the carboxylate (COO⁻) group [46–48]. Bands at 1377–1386 cm⁻¹ correspond to symmetric deformation of C-H (in CH₃) and C − N stretching, and those at 1058–1071 cm⁻¹ relate to primary and secondary –OH stretching [43–44]. FTIR spectroscopy identified the interaction modes and reactive groups affected by Cu²⁺ binding. The spectra of the loaded sorbent were similar to the pristine ones, with notable changes including decreased peak intensity and broadening rather than shifts in band positions. The binding mechanisms involved functional groups such as –OH, –NH, and –COOH. The changes observed are summarized as follows:

- A decrease in intensity for –OH and –NH peaks, with a blue shift from 3401 cm⁻¹ and 1062 cm⁻¹ to 3406 cm⁻¹ and 1071 cm⁻¹, indicating metal ion binding.
- A significant decrease in C = O stretching and amine bending vibrations at 1635 cm⁻¹.
- Changes in band width and intensity within 1650 –400 cm⁻¹, evidencing the involvement of these peaks (–NH, –OH, and –C = O or –COO⁻) in the binding mechanism.

#### XRD analysis

Figure 2c, d compares the XRD spectra of the various synthesized nanocomposites. The r-PG pattern (post-carbonization) shows a broad, disordered graphitic carbon peak (002) in the 2θ range of 23°-25°, with a d-spacing of ~ 3.73 Å, consistent with JCPDS 01-083-3674, indicating inter- and intra-molecular hydrogen bonds [47, 48]. The broad peak (002) nature indicates the amorphous character, arising from randomly oriented polycyclic aromatic carbon rings [5]. The XRD data for synthesized Cu⁰ nanoparticles reveal peaks at 43.2°, 50.23°, and 74.1°, corresponding to the (111), (200), and (220) planes, with d-spacings of 2.09 Å, 1.82 Å, and 1.28 Å, respectively. These angles and spacings are indicative of the face-centered cubic (FCC) structure of copper and space group Fm-3 m(No. 225) according to the Cu^0^ - JCPDS Number (01-071-4610(1) [26, 49]. In general, no impurity peaks (e.g., CuSO_4_, CuO) were detected, confirming the nanoparticles’ high purity. Notably, the XRD pattern of Cu25/HC reveals a weak diffraction peak at 75.47°, corresponding to trace CuO (JCPDS 00-001-1117). The observed peak broadening was primarily attributed to the nanoscale dimensions of the particles. Accordingly, the crystallite size was calculated using Scherrer’s equation (D = kλ/β cos θ), where λ = 1.5418 Å and k = 0.9. The average crystallite sizes of copper nanoparticles, estimated from the two most intense XRD diffraction peaks (namely, (200) and (220) reflections (Table S2)), increased progressively with Cu loading, yielding average sizes of 29.34 ± 4.26, 39.62 ± 2.47, and 54.79 ± 8.87 nm for Cu10/HC, Cu25/HC, and Cu50/HC, respectively. These values closely matched the SEM particle size ranges (25–35, 35–45, and 50–65 nm), confirming the consistency of the crystallographic and morphological analyses.

Notably, the XRD pattern of the spent Cu50/HC catalyst after the catalytic esterification reaction (Fig. 2d) exhibited a broad diffraction hump centered at approximately 20–27°, which is characteristic of amorphous carbonaceous materials and overlapped with the amorphous organic phase derived from the adsorbed esterification reactants and products. This broad feature is attributed to the disordered carbon structure and extensive hydrogen-bonding interactions among oxygenated organic species, in good agreement with the FTIR and XPS results and previous reports [50, 51]. Furthermore, comparison with the standard XRD pattern of monoclinic CuO (ICDD PDF No. 48-1548), which exhibits weak characteristic reflections at 2θ = 32.53° (110), 35.54° (− 111), 38.71° (111), 48.73° (− 202), 53.43° (020), 61.54° (− 113), 66.23° (− 311), 68.14° (220), and 72.35° (311), were detected in the spent catalyst, particularly the most intense (− 111) peak at 35.54° [52]. These results indicate that the copper nanoparticles largely retained their metallic crystalline structure with some CuO formation during the catalytic reaction. These results indicate good catalytic stability and reusability over several cycles.

#### Textural properties

The textural characteristics of all materials were evaluated using N₂ adsorption/desorption isotherms (Fig. 2e, f and Table S3). All materials exhibited similar Type II isotherm profiles with weak hysteresis loops, indicative of a wide pore size distribution characteristic of mesoporous materials. The specific surface area (SBET) followed the order: Cu25/HC (29.64 m²/g) > Cu10/HC (28.47 m²/g) > r-PG (28.07 m²/g) > Cu50/HC (14.94 m²/g). Corresponding total pore volumes were 0.099, 0.081, 0.069, and 0.066 cm³/g, respectively. Notably, increasing the copper loading from 10 to 25 mM resulted in a slight enhancement in surface area and pore volume compared to the pristine hydrochar. Improved texture results from Cu nanoparticle nucleation, which exfoliates the carbon matrix, creating added porosity and surface roughness [53]. In contrast, the Cu50/HC (50 mM) exhibited a marked reduction in S_BET_ (14.94 vs. 28.07 m².g^− 1^) and pore volume (0.066 vs. 0.069 cm³.g^− 1^), likely due to pore blockage from excessive copper nanoparticle deposition, which occludes micro- and mesopores [54]. The average pore size across all materials ranged from 2.0 to 2.2 nm, categorizing them as mesopores according to IUPAC standards (2–50 nm) [55].

#### X-ray photoelectron spectroscopy (XPS) analysis

**Fig. 3:**
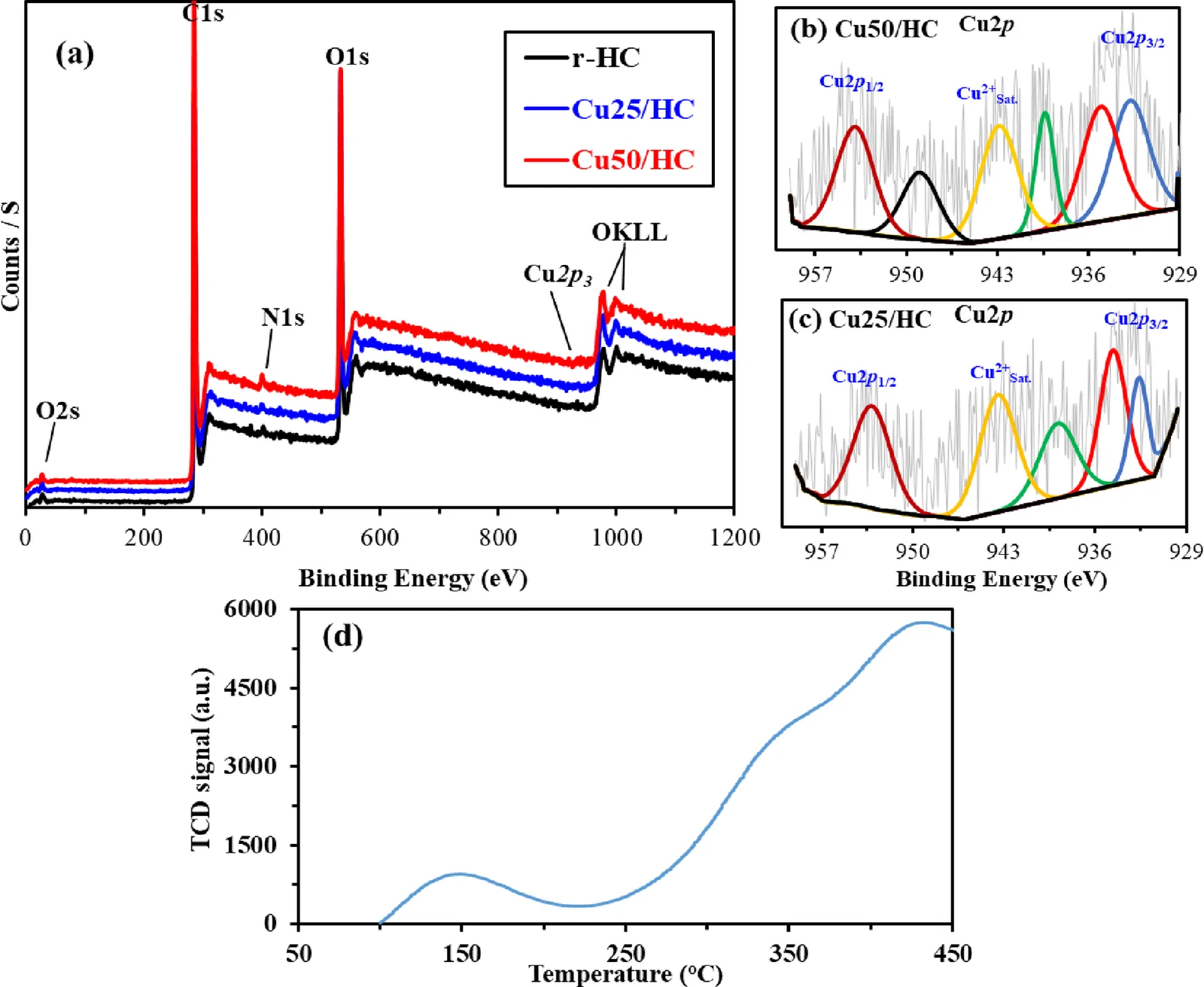
XPS survey spectra of r-PG, Cu25/HC, and Cu50/HC (**a**), HR-XPS Cu2*p* of Cu50/HC (**b**) and Cu25/HC (**c**), and the TPD of ammonia for Cu50/HC (**d**)

Full-scan XPS spectra of r-PG and the nanocomposites (Cu25/HC and Cu50/HC) (Fig. 3a and Table S4) reveal clear compositional modifications following copper incorporation. The primary elemental signals detected include C1s (approximately 285.4–286.7 eV), N1s (approximately 400.3–402.5 eV), and O1s (approximately 533.1–534.2 eV). After copper loading, a Cu2p_3/2_ signal appears at approximately 940.1 eV, accompanied by a weak CuLMM feature around 335–337 eV [53, 56]. The deconvoluted XPS core-level data for C1s, O1s, N1s, and Cu2p, including binding energies, atomic percentages, and the chemical assignments are provided in Table S3 in the Supplementary Information. To investigate the copper-related secondary phases, high-resolution Cu 2p core level XPS spectra were analyzed (see Fig. 3b, c)). The Cu valence states can be distinguished: the Cu 2p_1/2_ peak near 932.64 eV (Cu25/HC) and 932.82 eV (Cu50/HC) indicate Cu⁰ (3d¹⁰, 4s¹) and Cu⁺ in Cu₂O (3d¹⁰, 4s⁰), while the Cu 2p_3/2_ peak at approximately 935.5 eV is associated with Cu²⁺ in CuO (3d⁹, 4s⁰) [6]. The absence of a Cu 2p_1/2_ peak near 955.6 eV further confirms the low content of Cu²⁺ in the samples. Notably, the relative peak intensity of Cu⁺ 2p_3/2_ compared to Cu²⁺ 2p_3/2_ is high, with no satellite structure present. Strong satellite peaks, typically found between 940 eV and 945 eV for Cu²⁺ (Cu-3d⁹), are absent for Cu⁰ and Cu⁺ in Cu_2_O (3d^10^, 4s^0^) [57, 58]. The absence or negligible presence of Cu²⁺ satellites in the CuO analysis supports the conclusion that Cu⁺ is the more dominant oxidation state compared to Cu²⁺. Therefore, the higher intensity of the Cu⁺ 2p_3/2_ peak and the lack of satellite structure indicate that Cu⁺ is more prevalent than Cu²⁺. This dominance of Cu⁺, with its filled 3d orbitals (3d¹⁰, 4s⁰), may explain the absence of CuO secondary phases in both XRD and Cu-XPS results. Consequently, Cu(II) was effectively reduced to Cu⁺ and Cu⁰ on the surfaces of hydrochar, as discussed previously, likely due to the presence of H₂(g) and CO₂(g).

#### NH_3_-TPD measurements

The surface acidity of the Cu50/HC was analyzed using the ammonia temperature-programmed desorption (NH₃-TPD) method [59]. The ammonia desorption peaks (Fig. 3d and Table S3) were observed at 147 °C, 351 °C (as a shoulder), and 433 °C, indicating surface acidity with distinct weak, medium, and strong acid sites [60]. The initial low-temperature desorption peak at 147 °C (i.e., desorption features below 200 °C) corresponds to weakly adsorbed ammonia not fully purged, typically linked to Lewis acid sites or weakly bound ammonium species in Cu-based catalysts [60]. Notably, the ammonia desorption peak typically observed in the range of 190–210 °C—which primarily indicates ammonia adsorbed on Cu²⁺ ions [36, 61] was nearly absent in the TPD profile. This confirms that most copper was reduced to Cu⁰ or Cu⁺ during catalyst preparation, consistent with XRD and XPS results. The desorption features observed above 350 °C (351 °C and 433 °C) correspond to ammonia released from strong acid sites, with a total acid amount of 54.9 µmol/g. In Cu-CHA zeolite systems, traditionally assigned to Brønsted sites in zeolites, recent studies show these peaks can also arise from strongly bound NH₃ on Cu species, such as [Cu(I)NH₃]⁺ and [Cu(II)(NH₃)₄]²⁺ complexes [59]. The 351 °C peak matches linear [Cu(I)(NH₃)₂]⁺ decomposition. Given overlapping contributions from Brønsted/Lewis sites and Cu⁺-bound ammonia [62, 63], both peaks are best attributed to multiple site types rather than a single origin.

### Catalytic performance evaluation for oleic acid esterification

**Fig. 4:**
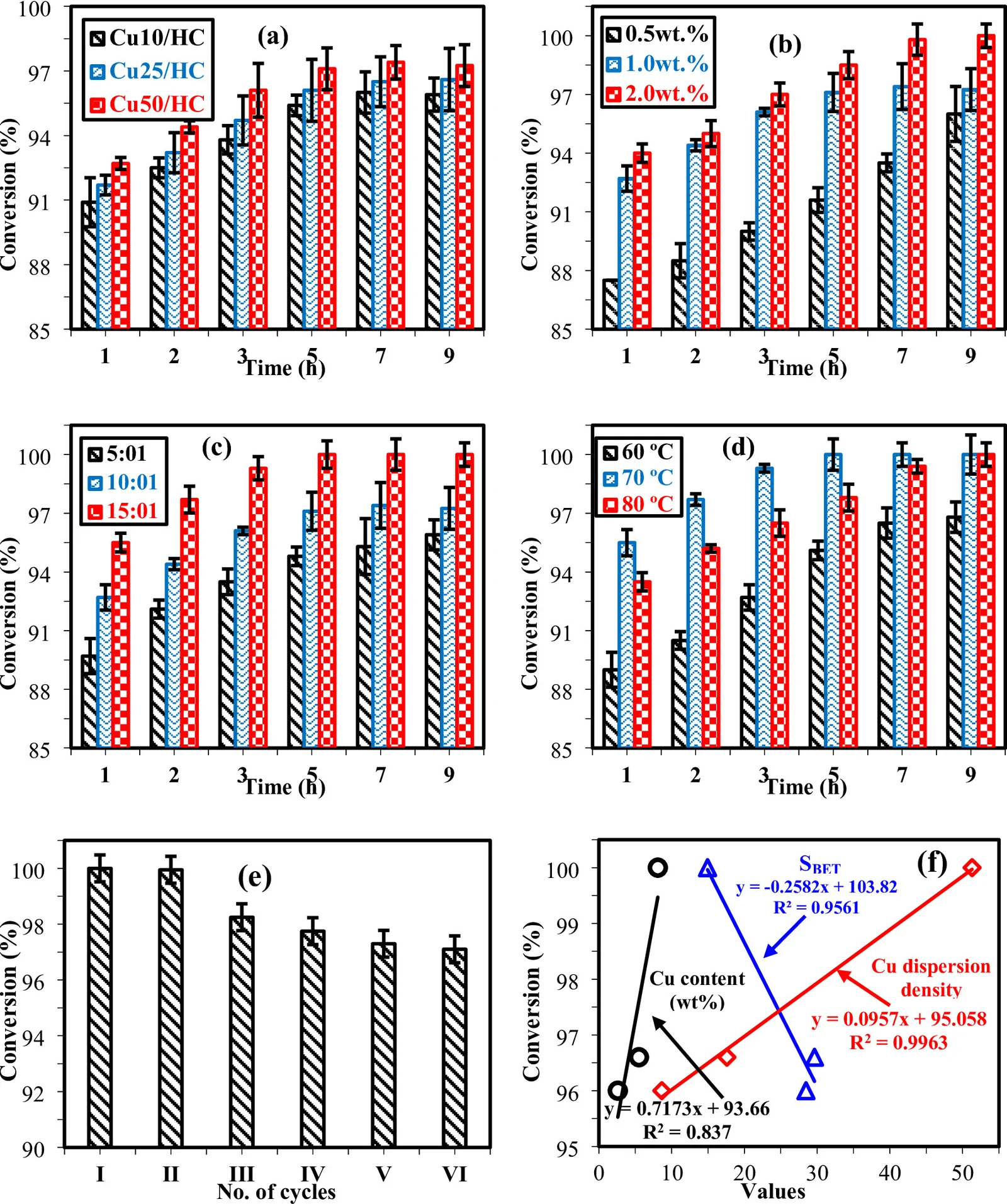
Single-factor optimization of the esterification reaction: (**a**) catalyst type, (**b**) catalyst loading (wt% relative to OA), (**c**) ethanol/OA ratio, (**d**) reaction temperature, (**e**) reusability performance of Cu₅₀/HC, and (**f**) structure–activity correlations of Cu/HC nanocomposites

The catalytic activity of the synthesized Cu/HC nanocomposites was evaluated for biodiesel production via the *esterification* of oleic acid (OA) with ethanol. A systematic single-factor optimization approach was employed to investigate the influence of reaction time (1 to 9 h), catalyst loading (1 to 5 wt%), ethanol-to-OA molar ratio (5:1 to 15:1), and temperature (60 to 80 °C) on OA conversion, as shown in Fig. 4.

#### Effect of reaction time

Figure 4a illustrates the time-dependent conversion profile over 1–9 h using 100 mg of catalyst (1 wt%), a 10:1 EtOH/OA molar ratio, and 70 °C. Catalytic conversion increased progressively with reaction time as prolonged contact between the reactants and catalyst active sites promoted esterification until equilibrium was approached [64–66]. After 7 h, OA conversion reached 93.8 ± 1.2%, 94.7 ± 0.9%, and 96.1 ± 0.7% for Cu10/HC, Cu25/HC, and Cu50/HC, respectively. Compared with pristine hydrochar (r-HC), which showed only 45.2 ± 2.1% conversion under identical conditions, demonstrating that copper loading significantly enhances activity. The superior catalytic performance of Cu50/HC is attributed to its higher copper loading, which provides a greater density of accessible Cu⁰/Cu⁺ Lewis acid sites and enhanced surface acidity (54.9 µmol/g; Table S3), thereby facilitating oleic acid activation and accelerating esterification. Extending the reaction beyond 7 h produced only marginal improvements in conversion, indicating that the reaction had approached equilibrium. At this stage, active-site saturation, reversible reaction behavior, and mass transfer limitations collectively restricted further conversion enhancement [65, 67]. Consequently, 7 h was selected as the optimum reaction time, with Cu50/HC exhibiting the highest catalytic efficiency and achieving an OA conversion of 97.4%.

##### Effect of catalyst loading

The influence of catalyst loading (0.5–2 wt% relative to oleic acid) on the esterification performance was evaluated using Cu50/HC under fixed reaction conditions (EtOH/OA = 10:1, 70 °C, and 9 h) (Fig. 4b). The OA conversion increased markedly with increasing catalyst loading up to 1 wt%, beyond which only a marginal improvement (2.4%) was observed at 2 wt%. This behavior indicates that 1 wt% (100 mg) provides a sufficient density of catalytically active sites to efficiently promote the reaction. The enhanced catalytic activity at lower loadings is attributed to the increased availability of accessible Lewis acid sites (Cu⁺/Cu⁰) and Brønsted acid sites (surface –OH groups), which facilitate oleic acid activation through protonation and subsequent nucleophilic attack by ethanol [66, 68–70]. Further increasing the catalyst loading (> 2 wt%) is unlikely to improve conversion because particle agglomeration reduces the exposed active surface area, while increased slurry viscosity and mass transfer limitations hinder the diffusion of reactants and products to and from the catalyst surface [64, 71]. Therefore, a catalyst loading of 1 wt% was identified as the optimum, providing complete conversion with minimal catalyst consumption and enhanced process economy.

##### Effect of ethanol-to-oleic acid molar ratio

The ethanol-to-OA molar ratio is a key parameter in esterification because the reaction is reversible and requires excess alcohol to shift the equilibrium toward ester formation in accordance with Le Chatelier’s principle. Using Cu50/HC (1 wt%) at 70 °C, the EtOH/OA molar ratio was varied from 5:1 to 15:1 (Fig. 4c). Increasing the ratio from 5:1 to 15:1 progressively enhanced OA conversion, reaching 97.4% at 15:1 after 5 h. This enhancement is attributed to the equilibrium shift toward ester production and the increased probability of effective collisions between ethanol and oleic acid molecules adsorbed on the catalyst surface, thereby promoting the forward reaction and improving catalytic efficiency [71–73]. However, ratios exceeding 15:1 were not examined because excessive ethanol may dilute the reaction medium, reduce the effective OA concentration, and competitively occupy active sites, limiting further enhancement in catalytic performance [64, 68, 73]. Therefore, an EtOH/OA molar ratio of 15:1 was identified as the optimum condition for achieving complete esterification.

##### Effect of reaction temperature

Temperature plays a dual role in esterification: it increases reaction kinetics but can also promote undesirable side reactions or alcohol evaporation. Using Cu50/HC (1 wt%) and a 15:1 EtOH/OA ratio, the reaction was conducted at 60, 70, and 80 °C (Fig. 4d). At 70 °C, complete OA conversion (97.4%) was achieved after 5 h, attributed to sufficient activation energy for the endothermic esterification reaction, which enhances molecular collision frequency and active site accessibility [66, 74]. Raising the temperature to 80 °C resulted in a significant decline in conversion, most likely due to ethanol evaporation (boiling point 78.4 °C), which reduces the effective alcohol concentration in the liquid phase and shifts the equilibrium back toward reactants. Lower temperatures (60 °C) yielded incomplete conversion due to insufficient kinetic energy. Therefore, 70 °C was selected as the optimal temperature, balancing high conversion efficiency with minimal energy consumption and alcohol loss. Increasing the reaction temperature enhanced esterification by overcoming the activation energy barrier and favoring the endothermic reaction. However, excessive temperatures promoted ethanol evaporation, reducing the effective reactant concentration and consequently limiting further conversion enhancement [66, 69, 70, 72].

Based on the above single-factor optimization, the optimal reaction conditions for OA esterification over Cu50 are: reaction time of 5 h, catalyst loading of 1 wt% (100 mg), ethanol-to-OA molar ratio of 15:1, and temperature of 70 °C. Under these conditions, the Cu50/HC catalyst achieved 97.4% OA conversion, demonstrating excellent catalytic performance for biodiesel production.

##### Catalyst reusability and stability

The reusability of the Cu50/HC catalyst for oleic acid esterification with ethanol was evaluated under optimized conditions (15:1 ethanol-to-OA molar ratio, 1 wt% catalyst, 70 °C, 5 h). After each cycle, the catalyst was recovered by centrifugation, washed with ethanol at 50 °C, and dried at 80 °C before reuse. The conversion efficiency decreased only marginally over six cycles (see Fig. 4e), retaining approximately 97.1% of initial activity, a loss of less than 3%. This high retained activity confirms the excellent chemical stability of the Cu50/HC catalyst. Notably, even partial oxidation of Cu⁰ to Cu⁺ (Cu₂O) or Cu²⁺ (CuO) occurs during repeated cycles; the resulting copper oxide species may still contribute to catalytic efficiency. As evidenced by XPS (Sect.  3.2), Cu⁺ was the dominant oxidation state in the fresh catalyst, and both Cu⁰ and Cu⁺ are known to be active for esterification. Also, the activation energy (73.6 kJ/mol) confirmed chemical control rather than diffusion limitations, implying sustained active site accessibility. The strong anchoring of copper nanoparticles onto the oxygen-functionalized hydrochar matrix minimizes leaching and sintering, while the slight activity loss likely arises from minor mass loss during recovery or adsorption of polar species. Overall, Cu50/HC demonstrates exceptional reusability, making it a promising waste-derived catalyst for sustainable biodiesel production.

The marginal decrease in catalytic activity (97.1% retention after six cycles) is attributed to surface oxidation of Cu⁰ to Cu⁺/Cu²⁺ species, as confirmed by XRD and FTIR analysis of the spent catalyst, along with active-site masking by adsorbed reaction intermediates and minor material loss during recovery. Notably, AAS analysis verified the absence of significant copper leaching, with the fresh catalyst containing 8.1 ± 0.3 wt% Cu and the recovered catalyst retaining 7.4 ± 0.22 wt% Cu after six cycles—representing a total copper loss of only 8.64% (0.7 wt%) and an average leaching rate of 1.44% per cycle. This limited leaching arises from strong metal–support interactions between Cu nanoparticles and oxygen-functionalized hydrochar groups. Consequently, the catalyst preserved 91.36% of its copper content and 97.1% of its activity, confirming excellent structural integrity, reusability, and long-term operational stability.

After the 6th reuse cycle, the FTIR spectra showed the Cu₅₀/HC catalyst retained its overall structure with minor noticeable surface chemical changes, confirming good structural stability. The broad O–H/N–H region and the principal carbonyl and C–O vibrations remain present, although with moderate shifts from 3124 cm⁻¹ to 3057 cm⁻¹ after reaction, caused by adsorption of reaction intermediates and products. Likewise, the C–H bands at 2931 and 2865 cm⁻¹ weakened, indicating removal of organic residues and formation of a new surface environment [75]. In the carbonyl region, the ester C = O band shifted from 1707 to 1704 cm⁻¹, with a new shoulder at 1689 cm⁻¹ indicating coordinated carboxylate species from oleic acid or ethyl oleate [76]. The asymmetric carboxylate band shifted from 1602 to 1589 cm⁻¹, while the symmetric band remained nearly unchanged (1441to 1440 cm⁻¹). The resulting decrease in Δν(COO⁻) (i.e., Δν = ν_as_ − ν_s_) from 161 to 149 cm⁻¹ suggests stronger carboxylate coordination to copper active sites after reaction, consistent with adsorbed intermediates or products [77, 78]. Additional changes occurred throughout the fingerprint region, where altered carboxylate vibrations and modified Cu–O bands reveal reconstruction of the copper surface. The bands at 1323, 1182, 1106, and 1049 cm⁻¹, assigned to C–H bending and C–O/C–O–C stretching vibrations of oxygen-containing surface functionalities [46], shifted to 1288, 1154, 1072, and 977 cm⁻¹, respectively, while new absorptions appeared at 891 and 812 cm⁻¹. The most notable changes appeared in the 900–400 cm⁻¹ region, corresponding to metal–oxygen vibrations. The appearance of a new 473 cm⁻¹ vibration together with shifts of the Cu–O bands (from 631 to 612 cm⁻¹ and 574 to 565 cm⁻¹) suggests partial surface oxidation of Cu nanoparticles to CuO (or Cu₂O/CuO mixed oxide) rather than complete oxidation or catalyst decomposition [52, 77]. Consequently, the FTIR results support that the Cu₅₀/HC catalyst maintains its structural integrity during esterification while experiencing only surface oxidation and adsorption-induced chemical modifications, which are typical for reusable copper-based heterogeneous catalysts.

#### Comparison with recently reported biomass-derived catalysts

The catalytic performance of the developed Cu₅₀/HC nanocomposite was benchmarked against recently reported biomass-derived and copper-based heterogeneous catalysts for biodiesel production (Table 1). The Cu₅₀/HC catalyst demonstrated exceptional activity, achieving 97.4% oleic acid conversion under mild conditions (70 °C, 5 h) with a remarkably low catalyst loading of just 1 wt%. This performance surpasses most reported systems, which typically require 2.5–8 wt% catalyst to reach conversions between 90.1% and 99.2%. The superior efficiency of Cu₅₀/HC is attributed to the synergistic interplay between highly dispersed Cu⁰/Cu⁺ nanoparticles and the oxygen-rich hydrochar matrix, providing both Lewis and Brønsted acid sites. This bifunctionality is a distinctive feature of the system, unlike single-function catalysts such as Fe₂(MoO₄)₃ [67] or sulfonated biochar catalysts [79]. Compared to copper-based systems such as CuO/UiO-66 [36], CuO/αFe₂O₃ [7, 14, 69], and Co@CuO [69], Cu₅₀/HC offers superior catalyst efficiency and reusability while avoiding expensive precursors. Compared to carbon-based systems such as sulfonated tea waste [79] and palm leaf hydrochar [5], Cu₅₀/HC achieves complete conversion with a significantly lower catalyst loading and maintains higher activity over more cycles.

**Table 1:** Comparative catalytic performance of Cu₅₀/HC and recently reported biomass-derived and copper-based heterogeneous catalysts

Catalyst	Preparation	Feedstock	Conversion (%)	Optimal Conditions	Reusability	Advantages	Disadvantages	Refs.
Fe₂(MoO₄)₃	Co-precipitation	OA + MeOH	92.50%	70 °C, 9:1 EtOH/OA, 5 wt% catalyst	90% (5 cycles)	Lewis acidic sites; sol-gel synthesis; reusable	Relatively high catalyst loading (5 wt%); moderate conversion	[67]
Sulfonated tea waste biochar (SO₃H-TDAC)	Sulfonation	OA + MeOH	95.40%	70 °C, 2.5 h, 7.5 wt% catalyst, 12:1 MeOH/OA	85% (5 cycles)	Waste-derived; high acid density (1.44 mmol/g); short reaction time	High catalyst loading (7.5 wt%); sulfonation requires concentrated H₂SO₄; methanol (toxic)	[79]
Rice husk ash/CuO/K₂CO₃	Wet impregnation	Sunflower oil	99.20%	65 °C, 2 h, 3 wt% catalyst, 18:1 MeOH/oil	88% (4 cycles)	Excellent yield (99.2%); short reaction time; waste-derived support (rice husk)	Complex multi-component synthesis; K₂CO₃ leaching risk; high methanol ratio (18:1)	[37]
Co@CuO	Co-precipitation	Wild oak oil	95.75%	59.73 °C, 2.5 h, 2.88 wt% catalyst, 10.08:1 MeOH/oil	92% (5 cycles)	Magnetic properties; low temperature; good reusability; novel feedstock	Cobalt toxicity; complex synthesis; moderate reusability	[69]
CuO/UiO-66	Solvothermal	Waste cooking oil	90.10%	80 °C, 4 h, 5 wt% catalyst	82% (4 cycles)	High surface area (MOF support); synergistic CuO-UiO-66 effect	Expensive MOF precursor; low conversion (90.1%); high temperature (80 °C); low reusability	[36]
Palm leaf hydrochar-SO₃H	Sulfonation	Palm FFA	93.56%	80 °C, 4 h, 4 wt% catalyst, 18:1 MeOH/PFAD	87% (5 cycles)	Waste-derived (palm leaf); hydrochar support; good stability	High catalyst loading (4 wt%); high temperature (80 °C); high methanol ratio (18:1); sulfonation step	[5]
CuO/αFe₂O₃	Co-precipitation	Waste cooking oil	94.27%	60 °C, 3 h, 2 wt% catalyst, 11:1 MeOH/oil	84% (5 cycles)	High surface area (334 m²/g); magnetic recovery; low catalyst loading (2 wt%)	Moderate conversion (94.27%); complex co-precipitation synthesis	[8]
NiFe₂O₄@g-C₃N₄	sol-gel technique	Waste cooking oil	98.83%	61.17 °C, 31 min, 2.85 wt% catalyst, 11.72:1 MeOH/oil	> 90% (7 cycles)	Very short reaction time (31 min); magnetic recovery; ultrasound-assisted; excellent yield	Complex synthesis (ultrasonication + pyrolysis); methanol (toxic); moderate catalyst loading	[69]
Cu₅₀/HC (This work)	Hydrothermal carbothermal	OA + EtOH	97.4%	70 °C, 5 h, 1 wt% catalyst, 15:1 EtOH/OA	97.1% (6 cycles)	Lowest catalyst loading (1 wt%); complete conversion; excellent reusability; waste-derived pomegranate peel support; ethanol (renewable); bifunctional acidity (Lewis + Brønsted); one-pot green synthesis.	Longer reaction time (5 h); high EtOH/OA ratio (15:1); needs validation with real feedstocks; ethanol recovery costs; limited to esterification (not transesterification yet)	This work

From a sustainability perspective, the Cu₅₀/HC catalyst is prepared from waste pomegranate peel-derived hydrochar, offering a cost-effective and environmentally benign approach to biomass valorization. It achieves complete oleic acid conversion under mild conditions using only 1 wt% catalyst and retains 97.1% of its initial activity after six reuse cycles, demonstrating excellent stability and recyclability. Nevertheless, several limitations should be acknowledged: the catalyst has been evaluated only for model oleic acid esterification, and validation with real high-FFA feedstocks remains necessary; the relatively high ethanol-to-oleic acid molar ratio (15:1) may affect large-scale economic feasibility due to alcohol recovery costs; and the longer reaction time (5 h) compared to some reported systems requires further optimization. From an industrial perspective, Cu₅₀/HC integrates seamlessly with existing biodiesel infrastructure, minimizing retrofitting costs. Its waste-derived support reduces raw material expenses, while adaptability to continuous flow enhances scalability. Ethanol serves as a renewable, less toxic alternative to methanol, improving the sustainability profile and positioning the catalyst as a cost-effective, environmentally compatible solution for commercial biodiesel production.

##### Structure–performance correlation

To elucidate the relationship between copper loading, textural properties, and catalytic performance, via the key characteristics, copper loading, textural properties, the Cu dispersion density was calculated as the bulk Cu content (wt%) per unit BET surface area (m² g⁻¹), using the Cu contents determined by AAS (2.6 ± 0.2, 5.5 ± 0.4, and 8.1 ± 0.3 wt% for Cu₁₀/HC, Cu₂₅/HC, and Cu₅₀/HC, respectively). In addition, the symbolic surface Cu density, expressed as the number of Cu atoms per nm² of S_BET_, was estimated to provide a quantitative measure of the accessible active-site concentration. It should be noted that the previously reported concept of amine density is not applicable here, as the present catalysts contain Cu species rather than amine functionalities. As summarized in Table 2, the Cu dispersion density increased significantly from 0.091 for Cu₁₀/HC to 0.186 for Cu₂₅/HC and 0.542 for Cu₅₀/HC, corresponding to a 5.94-fold enhancement. Likewise, the Cu surface atom density reached 51.3 atoms nm⁻² for Cu₅₀/HC, approximately 2.92 and 5.93 times higher than those of Cu₂₅/HC (17.6 atoms nm⁻²) and Cu₁₀/HC (8.65 atoms nm⁻²), respectively. The substantially greater concentration of surface-exposed Cu species generates a higher density of accessible Lewis acid sites, thereby promoting oleic acid adsorption and activation and resulting in complete (97.4%) conversion.

To quantitatively elucidate the structure–activity relationships governing catalytic performance, correlation analyses were performed between key physicochemical parameters and oleic acid conversion (Fig. 4f). The Cu dispersion density (Cu Atoms/nm², as *x* axis), versus the conversion percent (i.e., *conversion* (%) as *y axis*; increases from 96.0% (Cu10/HC), 96.6% (Cu25/HC), and 97.4% (Cu50/HC) exhibited an excellent linear correlation with conversion (*x* = 0.0957×(*y)* + 95.058; R^2^: 0.996). This strong correlation identifies surface Cu concentration as the primary descriptor of catalytic activity. In contrast, bulk copper content (wt%, as *x* axis) analysis showed a notably weaker correlation (*x* = 0.7173×(*y)* + 93.66; R^2^: 0.837), indicating that not all Cu atoms present in the bulk are equally accessible or catalytically relevant. Most significantly, BET surface area (S_BET_, in *x axis*) exhibited an inverse correlation with conversion (*x* = -0.2582×(*y)* + 103.82; R^2^: 0.956). The negative slope confirms that higher surface area does not necessarily translate to superior performance. This counterintuitive behavior is attributed to progressive pore blockage by Cu nanoparticles at higher loadings, reducing the surface area from 28.47 to 14.94 m² g⁻¹ while increasing the surface Cu dispersion density to 51.3 atoms nm⁻². Despite its lowest surface area, Cu₅₀/HC achieved complete oleic acid conversion, demonstrating that active Cu site density, rather than surface area, is the primary determinant of catalytic performance.

**Table 2:** Correlation between copper loading, textural properties, Cu dispersion density, and catalytic performance of Cu/HC nanocomposites

Sample	Cu content^a^ (wt%)	S_BET_ (m²/g)	Cu dispersion density^b^	Copper (Atoms/nm²)	(Conversion, %)	Interpretation
Cu10/HC	2.6 ± 0.2	28.47	0.0913	8.65	96.00%	Low Cu density; limited active sites
Cu25/HC	5.5 ± 0.4	29.64	0.1856	17.6	96.60%	Moderate Cu density; improved activity
Cu50/HC	8.1 ± 0.3	14.94	0.5422	51.3	97.4%	Highest Cu density; best performance

N.B.: ^a^: by AAS, ^b^: wt%/(m²/g)

#### Kinetic and thermodynamic study

Understanding the kinetics and thermodynamics of the esterification reaction is essential for reactor design, process optimization, and evaluating the practical feasibility of the Cu50/HC catalyst for biodiesel production. The esterification of oleic acid (OA) with ethanol (EtOH) over the Cu50/HC catalyst proceeds according to the following reversible reaction:

3 $$ \begin{gathered} Oleic{\text{ }}acid{\text{ }}\left( {OA} \right){\text{ }} + {\text{ }}Ethanol{\text{ }}\left( {EtOH} \right) \hfill \\ \rightleftharpoons Ethyl{\text{ }}Oleate{\text{ }}\left( {EO} \right){\text{ }} + {\text{ }}Water{\text{ }}\left( W \right)~ \hfill \\ \end{gathered} $$

##### Kinetic analysis: pseudo-first-order model

**Fig. 5:**
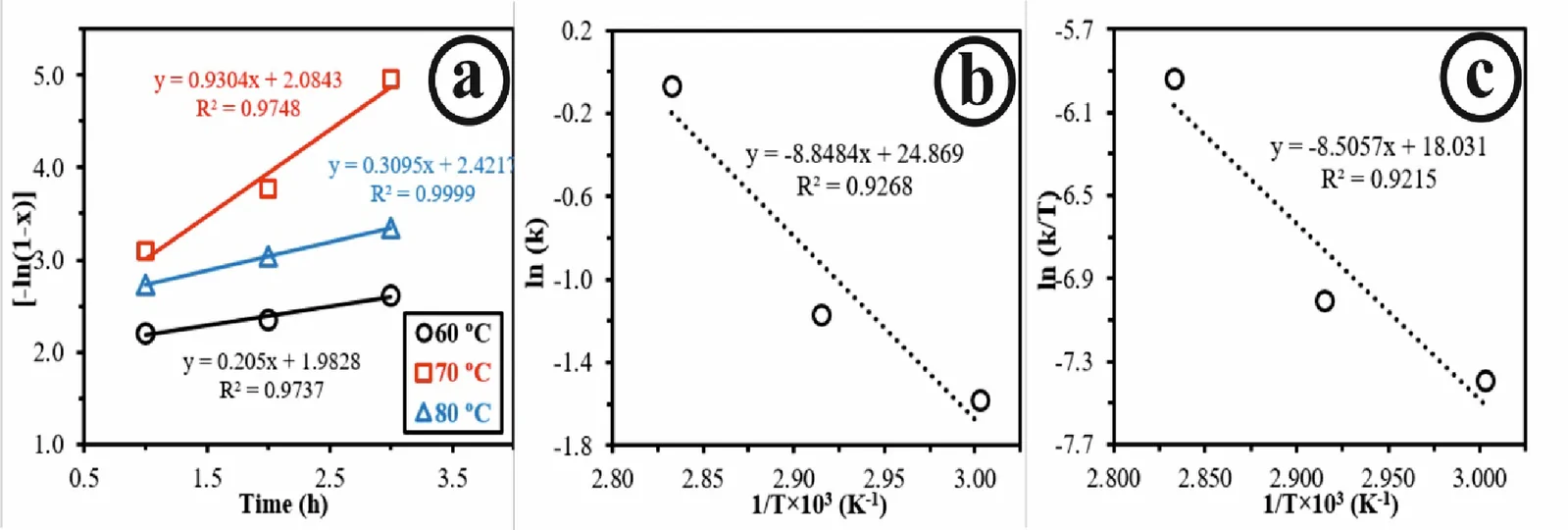
Kinetic and thermodynamic parameters of oleic acid esterification (Cu50/HC): **a** pseudo-first-order rate plot, **b** Arrhenius plot, and **c** Eyring–Polanyi plot (1/T vs. ln k/T)

Given that the optimal ethanol-to-oleic acid molar ratio was determined to be 15:1, ethanol is present in significant excess relative to OA. Under this condition, the concentration of ethanol remains essentially constant throughout the reaction, and the forward reaction rate depends solely on the concentration of oleic acid. This allows the esterification to be approximated as a pseudo-first-order reaction with respect to OA [74, 79]. The integrated rate law for a pseudo-first-order reaction is expressed as:

4 $$- {\mathrm{ln}}\left( {{\mathrm{1}}\, - \,{\mathrm{x}}} \right)\,=\,{\text{kt }}$$

where ‘x’ represents the fractional conversion of oleic acid at time ‘t’, and ‘k’ is the apparent first-order rate constant (min⁻¹). As shown in Fig. 5a and summarized in Table 3, a linear relationship between − ln(1 − x) and reaction time was observed at all three temperatures (333, 343, and 353 K). The high correlation coefficients (R² > 0.97) confirm that the pseudo-first-order model adequately describes the esterification kinetics over the Cu50/HC catalyst. The rate constant ‘k’ increased substantially with temperature: from 0.205 min⁻¹ at 333 K (60 °C) to 0.3095 min⁻¹ at 343 K (70 °C), and further to 0.9304 min⁻¹ at 353 K (80 °C). This nearly five-fold increase in ‘k’ upon raising the temperature by 20 °C demonstrates the strong temperature sensitivity of the reaction, which is characteristic of chemically controlled processes.

##### Activation energy determination

The temperature dependence of the rate constant was analyzed using the Arrhenius equation [37]:

5 $$\:lnk=ln{A}_{0}-{E}_{a}RT$$

where *A₀* is the pre-exponential factor (frequency factor),* E*_*a*_ is the activation energy (kJ·mol⁻¹), *R* is the universal gas constant (8.314 × 10⁻³ kJ·mol⁻¹·K⁻¹), and *T* is the absolute temperature (K). From the Arrhenius plot of ln *k* versus 1/*T*, the activation energy was calculated to be 73.6 kJ·mol⁻¹. This value is highly instructive for several reasons. First, activation energies for esterification reactions typically range from 30 to 80 kJ·mol⁻¹. The calculated * E*_*a*_ of 73.6 kJ·mol⁻¹ falls at the upper end of this range, indicating that the Cu50 catalyst requires a moderate to high energy input to initiate the reaction. Second, activation energies above approximately 50 kJ·mol⁻¹ are generally considered indicative of a chemically controlled reaction rather than a diffusion-controlled one [79]. Diffusion-controlled processes typically exhibit * E*_*a*_ values below 20–30 kJ·mol⁻¹. Therefore, the observed * E*_*a*_ of 73.6 kJ·mol⁻¹ confirms that the esterification over Cu50/HC is governed by the intrinsic chemical reaction kinetics at the active sites, not by mass transfer limitations. This is consistent with the experimental observation that increasing temperature dramatically enhances the conversion rate.

##### Thermodynamic parameters via eyring–polanyi theory

To assess thermodynamic parameters such as enthalpy (∆H), entropy (∆S), and Gibbs free energy (∆G), experiments were conducted within a temperature range of 60–80 °C, based on the Eyring–Polanyi equation [80]:

6 $${\mathrm{ln}}\left( {{\mathrm{k}}/{\mathrm{T}}} \right){\text{ }}={\text{ }}\left( {\Delta {\mathrm{S}}/{\mathrm{R}}} \right){\text{ }} - {\text{ }}\left( {\Delta {\mathrm{H}}/{\mathrm{RT}}} \right)\,+\,{\mathrm{ln}}\left( {{{\mathrm{k}}_{\mathrm{b}}}/{\mathrm{h}}} \right)$$

where k is the rate constant at temperature T, and k_b_ and *h* are the Boltzmann and Planck constants, respectively. By plotting ln(k/T) against 1/T (Fig. 5c), the slope yields -ΔH/R and the intercept provides ΔS/R. Meanwhile, the values of the Gibbs energy change (ΔG, kJ mol^− 1^) at different temperatures were determined by using Eq. (4)

7 $$\Delta {\mathrm{G}} = \Delta {\mathrm{H}}{-}{\mathrm{T}}\Delta {\mathrm{S}} $$

The resulting thermodynamic parameters are summarized in Table 3. The determined values for ΔH and ΔS in biodiesel production via the esterification of oleic acid using the Cu50 catalyst were 70.7 kJ/mol and − 0.048 kJ/mol.K, respectively. The calculated ΔG values were 86.6, 87.1, and 87.5 kJ/mol for reactions conducted at 60, 70, and 80 °C, respectively. The positive ΔH and ΔG indicate that the esterification process is endothermic and non-spontaneous, requiring an energy input. The negative ΔS suggest low entropy during the process, indicating that energy is primarily directed toward completing the esterification reaction to produce biodiesel [79, 81].

The fact that ΔH is positive means that more energy, in the form of heat, is needed to break through the activation barrier and encourage the production of the activated complex [81]. A decrease in disorder during the creation of the transition state is suggested by the negative value of ΔS (− 0.048 kJ·mol⁻¹·K⁻¹). The associative reaction mechanism states that a negative entropy of activation results from the reactant molecules joining together to produce a more ordered activated complex than the original reactants in the ground-state reactants. As a result, the observed negative ΔS values are consistent with a higher degree of molecular organization in the transition state compared to the reactants. Negative ΔS values are typical for bimolecular reactions where two reactants combine into a single activated complex [37]. The relatively small magnitude (− 0.048 kJ·mol⁻¹·K⁻¹) suggests moderate ordering, which is reasonable for a liquid-phase esterification involving a bulky fatty acid molecule [37, 80]. The positive Gibbs free energy of activation (ΔG = 86.6–87.5 kJ·mol⁻¹) confirms that activated complex formation is non-spontaneous. ΔG increases slightly with temperature (86.6 kJ·mol⁻¹ at 333 K to 87.5 kJ·mol⁻¹ at 353 K), driven by the negative ΔS term (ΔG = ΔH − TΔS). As temperature rises, −TΔS becomes more positive, raising ΔG. Thus, while higher temperatures increase the rate constant kinetically, they make the activation step less thermodynamically favorable [82]. Nevertheless, the kinetic benefit dominates, evidenced by higher conversion at 70 °C versus 60 °C.

The activation parameters of Cu50/HC (ΔG = 86.6 kJ mol⁻¹, ΔH = 70.7 kJ mol⁻¹, ΔS = − 0.048 kJ mol⁻¹ K⁻¹) fall within the range reported for other heterogeneous catalysts, while the relatively less negative ΔS suggests a less ordered transition state compared to ZIF-67@Fe₃O₄ (ΔS = − 172 J mol⁻¹ K⁻¹) [83], consistent with the flexible bifunctional acid sites of the hydrochar-supported Cu catalyst. These thermodynamic characteristics are consistent with earlier research on the esterification of oleic acid with alcohol over a range of heterogeneous catalysts, which found that the reaction was endergonic, non-spontaneous, and endothermic (Table 4).

**Table 3:** Pseudo-first-order rate constants and thermodynamic parameters for oleic acid esterification over Cu50/HC

Type	Pseudo-first-order rate constants				Thermodynamic parameters		
Temp. (K)	k (min⁻¹)	*R*²	E_a_ (kJ mol⁻¹)	A₀ (min⁻¹)	ΔH (kJ mol⁻¹)	ΔS (kJ mol⁻¹ K⁻¹)	ΔG (kJ mol⁻¹)
333	0.21	0.97	73.6	1.00 × 10²	70.7	-0.048	86.6
343	0.31	0.99					87.1
353	0.93	0.97					87.5

N.B.: k = pseudo-first-order rate constant determined from –ln(1–x) = *kt*. Both ΔH and ΔS are constant values

**Table 4:** Comparison of thermodynamic parameters of oleic acid esterification over different catalysts

Catalyst	ΔG, kJ mol⁻¹	ΔH, kJ mol⁻¹	ΔS, kJ mol⁻¹K^− 1^	Refs.
Spent alkaline batteries	94.666	19.274	N/A	[66]
Sulfonated biochar	95.17	27.6	-0.19	[79]
ZIF-67@Fe_3_O_4_	88.44	32.02	-172	[83]
Na_2_O/Ba-chitosan	79.77	77	-65.065	[84]
Cu50/HC	86.6	70.7	-0.048	This Work

#### Proposed catalytic mechanism of oleic acid esterification over Cu₅₀/HC

**Fig. 6:**
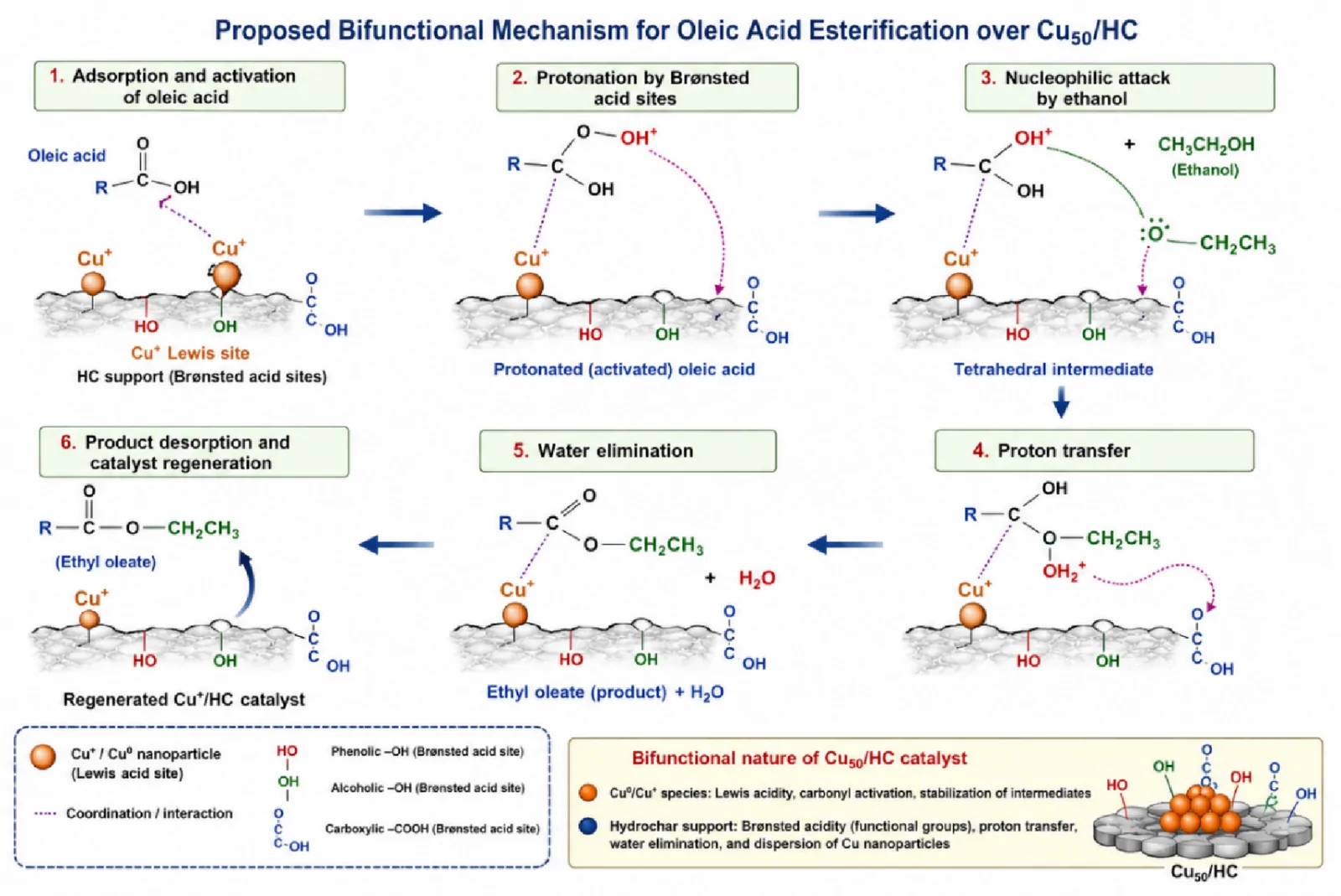
Proposed bifunctional catalytic mechanism for oleic acid esterification over Cu50/HC nanocomposite. Cu⁺ species act as Lewis acid sites that coordinate and activate the carbonyl group of oleic acid, while oxygen-containing functional groups on the hydrochar matrix provide Brønsted acidity for proton transfer. The synergistic interaction promotes ethanol attack, tetrahedral intermediate formation, water elimination, and catalyst regeneration

To elucidate the exceptional esterification performance of Cu₅₀/HC, we propose a bifunctional catalytic mechanism grounded in physicochemical characterization and kinetic data (Fig. 6). This mechanism hinges on the synergistic interplay between Lewis acidic Cu⁰/Cu⁺ species and Brønsted acidic oxygen-containing functional groups inherent to the hydrochar support [37]. The catalytic cycle commences with the adsorption of OA onto the catalyst surface, wherein the carbonyl oxygen coordinates with exposed Cu⁺ sites (R−COOH + Cu^+^→ R−COOH⋯Cu^+^, where R=C_17_H_33_). This Lewis acid–base interaction enhances the electrophilicity of the carbonyl carbon, rendering it more susceptible to nucleophilic attack by ethanol—a pathway consistent with established Cu-based esterification systems. Concurrently, surface carboxylic, phenolic, and hydroxyl moieties on the hydrochar matrix protonate the carbonyl oxygen (R − C(= O) − OH → R − C(= OH^+^) − OH), further polarizing the C = O bond and diminishing the activation energy barrier for ester formation. Subsequently, nucleophilic ethanol attacks the activated carbonyl carbon, generating a tetrahedral intermediate (R − C(= OH^+^) − OH + CH_3_CH_2_OH → R − C(OH)(OCH_2_CH_3_) − OH). Proton transfer within this intermediate converts the hydroxyl group into a proficient leaving group (R − C(OH)(OEt) − OH → R − C(OEt)(OH_2_^+^) − OH), facilitating dehydration and the eventual release of ethyl oleate (R − C(OEt)(OH_2_^+^) − OH → R − C(= O) − OEt +H_2_O). The ester product desorbs (R-COOCH_2_CH_3_ + Cu^+^), regenerating both Lewis-active Cu⁺ and Brønsted acid sites for successive catalytic cycles.

This mechanistic proposal is robustly substantiated by characterization. XPS corroborates the coexistence of Cu⁰ and Cu⁺—acknowledged Lewis acid centers for carbonyl activation. FTIR identifies abundant oxygenated surface functionalities, while NH₃-TPD confirms accessible acidic sites. The superior activity of Cu₅₀/HC over Cu₁₀/HC and Cu₂₅/HC is attributable to its higher density of exposed Cu sites and the amplified synergy between copper species and surface acid groups. This cooperative effect optimizes reactant activation, stabilizes intermediates, and accelerates the overall esterification, manifesting in remarkable oleic acid conversion. Our findings align with prior reports on Cu-catalyzed esterification, wherein Lewis acidity drives carbonyl polarization and Brønsted acidity expedites proton transfer and dehydration, collectively ensuring high catalytic efficiency [6, 7, 10, 36].

## Conclusion

A cost-effective, sustainable nanocomposite catalyst comprising Cu⁰ nanoparticles supported on pomegranate peel-derived hydrochar was synthesized via a one-pot hydrothermal carbothermal reduction method. XRD confirmed crystalline Cu⁰ with a face-centered cubic (FCC) structure and spherical morphology (25–65 nm), consistent with SEM analysis. Catalytic activity increased with copper loading; Cu50/HC achieved the highest oleic acid conversion (97.4%) under optimized conditions (70 °C, 5 h, 1.0 wt% catalyst, 15:1 ethanol/OA molar ratio), followed by Cu25/HC (96.6%) and Cu10/HC (96.0%). Complete OA conversion was attained with Cu50/HC. The Cu dispersion density (Cu atoms/nm²) exhibited an excellent linear correlation with conversion (R² = 0.996), identifying surface Cu concentration as the primary activity descriptor, compared to bulk copper content (R² = 0.837) and S_BET_ (R² = 0.956), which showed weaker or inverse correlations. Kinetic analysis revealed a pseudo-first-order model with activation energy of 73.6 kJ mol⁻¹, while thermodynamic parameters (ΔH‡ = 70.7 kJ mol⁻¹, ΔS‡ = − 48 J mol⁻¹ K⁻¹, ΔG‡ = 87.1 kJ mol⁻¹) confirmed a kinetically hindered but chemically controlled process. The bifunctional catalytic mechanism, combining Lewis acidity from Cu⁺ species and Brønsted acidity from hydrochar oxygen functionalities, was elucidated and supported by XPS, FTIR, and NH₃-TPD analyses. Notably, the catalyst retained 97.1% of its initial activity after six cycles, with only 8.64% total copper loss (0.7 wt%) and an average leaching rate of 1.44% per cycle, demonstrating excellent structural integrity and reusability. These results position the Cu50/HC nanocomposite as an economical, efficient, and sustainable catalyst for biodiesel production.

However, the present study is limited to the esterification of free fatty acids using oleic acid as a model compound. Evaluation of catalyst performance toward transesterification of triglyceride-rich feedstocks and validation with real high-FFA waste oils will be addressed in future studies. Furthermore, optimization of reaction conditions to reduce the ethanol-to-OA molar ratio and reaction time, along with techno-economic assessment for industrial scaling, represent promising directions for future research.

## Supplementary Information

Below is the link to the electronic supplementary material.

Supplementary Material 1.

## Data Availability

All data are given in the manuscript and Supplementary Information Section.
