# Supplementary material for: Pomegranate peel-derived Cu/hydrochar nanocomposites: a sustainable catalyst for biodiesel production via oleic acid esterification
Source: BMC Chem. 2026 Oct 6;20(1):201. doi: 10.1186/s13065-026-01885-z (PMC13644243; doi:10.1186/s13065-026-01885-z)
Supplement: Supplementary file 1 [file 13065_2026_1885_MOESM1_ESM.docx]

**Supplementary Information Section**

**Pomegranate Peel-Derived Cu/Hydrochar Nanocomposites: A Sustainable Catalyst for Biodiesel Production via Oleic Acid Esterification**

**Amal A. Alkahlawy ^a, *^, Delvin Aman^a^, Ahmed A. Galhoum^b^, Tamer Zaki ^a^**

^a^ Petroleum Refining Department, Egyptian Petroleum Research Institute (EPRI), 1 Ahmed El Zomour St., Nasr City, PO Box 11727, Cairo, Egypt

^b^ Nuclear Materials Authority, P.O. Box 530, El-Maadi, Cairo, Egypt.

**Keywords:** Pomegranate peel, Hydrothermal treatment, Cu/hydrochar nanocomposite, Biodiesel, Catalytic esterification, Oleic acid.

**____________________________________________**

***Corresponding author: E-mail:** amal_kahlawy@ yahoo.com(Amal A. Alkahlawy), [delvin.aman@epri.sci.eg](mailto:delvin.aman@epri.sci.eg) (Delvin Aman), g[alhoum_nma@yahoo.com](mailto:alhoum_nma@yahoo.com) (Ahmed Galhoum), and [tamer_zaki_sharara@epri.sci.eg](mailto:tamer_zaki_sharara@epri.sci.eg) (Tamer Zaki).

**Section A. Results and discussion**

**A.1. Characterization Section**

Fig. S1. EDX analysis for the r-PG (a), Cu10/HC (b), Cu25/HC (c), and Cu50/HC (d).

**Table S1.** FTIR assignments of pristine PG and after Cu/HC with different concentrations of loading.

| **Assignment** | **PG-powder** | | **r-PG** | | **Cu10/HC** | | **Cu25/HC** | | **Cu50/HC** | | **Ref.** | |
| --- | --- | --- | --- | --- | --- | --- | --- | --- | --- | --- | --- | --- |
| –OH overlapped with –NH str. | 3419 | 3422 | | 3421 | | 3393 | | 3429 | | [1, 2] | |  |
| C-H str. asym. (CH_2_) | 2920 | 2921 | | 2917 | | 2919 | | 2922 | | [3] | |  |
| C-H str. sym. (CH_2_) | --- | 2849 | | 2849 | | 2856 | | 2854 | |  |  |  |
| C=O (Carboxylic acid) | 1726 | 1704 | | 1699 | | 1703 | | 1704 | | [3] | |  |
| COO^-^ sym. str., overlapped with –C=C, -C=N, str. and N−H bend. | 1628 | 1617 | | 1620 | | 1613 | | 1617 | | [4, 5] | |  |
| COO‐ sym. str., CH_2_ bend | 1431 | 1438 | | 1434 | | 1432 | | 1438 | | [48] | |  |
| C-H sym. bend., C-N str. | 1373 | 1336 | | 1351 | | 1379 | | 1338 | | [2] | |  |
| C−O−C asym. str., C−O str, C−N str. Skeletal C-O str. | 1216 | 1191 | | 1190 | | 1162 | | 1193 | | [5] | |  |
| C‐O str., -OH 1^o^ alc., | 1042 | 1055 | | 1053 | | 1053 | | 1078, 1054 | | [3] | |  |
| C‐H out‐of‐plane bend, ‐NH twist  M-O str. |  | 756 | | 758 | | 859, 767 | | 752 | | [5] | |  |
|  |  | 617, 518 | | 629,  514 | | 664 | | 609 | |  |  |  |
| M-O str. | 485 |  | | 428 | | 492 | | 492 | |  | |  |

**Table S2.** Crystallite sizes determined from XRD ((200) and (220) planes) and SEM analysis.

| **Sample** | **Crystallite size from (200) plane (nm)** | **Crystallite size from (220) plane (nm)** | **Average crystallite size (nm)** | **SEM particle size range (nm)** |
| --- | --- | --- | --- | --- |
| Cu10/HC | 25.08 | 33.59 | 29.34 ± 4.26 | 25–35 |
| Cu25/HC | 37.15 | 42.08 | 39.62 ± 2.47 | 35–45 |
| Cu50/HC | 63.65 | 45.92 | 54.79 ± 8.87 | 50–65 |

**Table S3.** Textural characteristics of hydrochar and Cu/HC with different concentrations loading.

| **Samples** | **S_BET_^a^ (m²/g)** | **Pore Volume^b^ (cm^3^/g)** | **Pore Size^c^ (nm)** | **Acid amount^d^ (µmol/g)** |
| --- | --- | --- | --- | --- |
| **Hydrochar** | 28.072 | 0.069 | 2.21 | **---** |
| **Cu10/HC** | 28.469 | 0.081 | 2.21 | --- |
| **Cu25/HC** | 29.639 | 0.099 | 2.01 | --- |
| **Cu50/HC** | 14.935 | 0.066 | 2.01 | 54.9 |

^a^: BET surface area, ^b^: Total pore volume at maximum *P*/*P^o^*, ^C^: Pore size distribution based on BJH, ^d^: Total acid amount measured by ammonia-TPD.

**Table S4.** Assignment of core-level signals: binding energies, atomic fractions (%), and chemical assignment.

| **Name** | **r-HC** | | **Cu25/HC** | | **Cu50/HC** | | **Chemical Assignment** |
| --- | --- | --- | --- | --- | --- | --- | --- |
|  | **Peak BE (eV)** | **Atomic %** | **Peak BE (eV)** | **Atomic %** | **Peak BE (eV)** | **Atomic %** |  |
| **C1s** | 284.54 | 68.56 | 284.31 | 68.19 | 284.33 | 77.23 | C–C / C=C (sp² carbon, graphitic) |
|  | 286.19 | 14.94 | 285.94 | 12.55 | 285.96 | 8.75 | C–O / C–OH (hydroxyl / ether) |
|  | 287.33 | 16.5 | 286.99 | 19.26 | 287.32 | 14.03 | C=O / O–C–O (carbonyl / carboxyl) |
| **O1s** | 531.42 | 21.14 | 531.52 | 29.45 | 531.11 | 25.96 | C=O (carbonyl / carboxyl oxygen) |
|  | 532.67 | 52.56 | 532.32 | 27.36 | 532.94 | 19.38 | C–OH / C–O–C (hydroxyl / ether oxygen) |
|  | 533.33 | 26.3 | 532.77 | 43.19 | 532.45 | 54.67 | O–C=O / adsorbed H₂O (carboxylate / moisture) |
| **N1s** | 400.38 | 100 | 399.85 | 100 | 399.78 | 100 | N–H / C–N (amine / imine / pyrrolic N) |
| **Cu2p** | — | — | 932.64 | 11.01 | 932.82 | 19.97 | Cu⁰ / Cu⁺ 2p₃/₂ (metallic Cu / Cu₂O) |
|  | — | — | 934.63 | 21.6 | 935.06 | 19.9 | Cu²⁺ 2p₃/₂ (CuO) |
|  | — | — | 938.87 | 17.04 | 939.36 | 10.03 | Cu²⁺ satellite (CuO, shake-up) |
|  | — | — | 943.45 | 26.74 | 942.87 | 19.72 | Cu²⁺ satellite (CuO, shake-up) |
|  | — | — | — | — | 948.9 | 11.75 | Cu²⁺ satellite (CuO, shake-up) |
|  | — | — | 953.12 | 23.62 | 953.9 | 18.63 | Cu 2p₁/₂ (Cu⁰/Cu⁺/Cu²⁺) |

**References**

[1] A. Benettayeb, A. Morsli, K.Z. Elwakeel, M.F. Hamza, and E. Guibal. Recovery of Heavy Metal Ions Using Magnetic Glycine-Modified Chitosan—Application to Aqueous Solutions and Tailing Leachate. Applied Sciences, 11 (2021) <https://doi.org/10.3390/app11188377>.

[2] A.S. Morshedy, A.A. Galhoum, A. Aleem H. Abdel Aleem, M.T. Shehab El-din, D.M. Okaba, M.S. Mostafa, H.I. Mira, Z. Yang, and I. E.T.- El-Sayed. Functionalized aminophosphonate chitosan-magnetic nanocomposites for Cd(II) removal from aqueous solutions: Performance and mechanisms of sorption. Applied Surface Science, 561 (2021) 150069. DOI: <https://doi.org/10.1016/j.apsusc.2021.150069>.

[3] J. Coates. Interpretation of infrared spectra: Apractical Approach. In Encyclopedia of analytical chemistry. 2000: John Wiley & Sons Ltd, Chochester. 10815-10837.

[4] A.A. Galhoum, E.A. Elshehy, D.A. Tolan, A.M. El-Nahas, T. Taketsugu, K. Nishikiori, T. Akashi, A.S. Morshedy, and E. Guibal. Synthesis of polyaminophosphonic acid-functionalized poly(glycidyl methacrylate) for the efficient sorption of La(III) and Y(III). Chemical Engineering Journal, 375 (2019) 121932. DOI: <https://doi.org/10.1016/j.cej.2019.121932>.

[5] M.F. Hamza, A.E. Mubark, Y. Wei, T. Vincent, and E. Guibal. Quaternization of Composite Algal/PEI Beads for Enhanced Uranium Sorption-Application to Ore Acidic Leachate. Gels, 6 (2020) 12. DOI: <https://doi.org/10.3390/gels6020012>.
